# Innovative Cosmeceutical Ingredients: Harnessing Selenosugar-Linked Hydroxycinnamic Acids for Antioxidant and Wound-Healing Properties

**DOI:** 10.3390/antiox13060744

**Published:** 2024-06-20

**Authors:** Giovanna Cimmino, Mauro De Nisco, Simona Piccolella, Claudia Gravina, Silvana Pedatella, Severina Pacifico

**Affiliations:** 1 Department of Environmental, Biological and Pharmaceutical Sciences and Technologies, University of Campania “Luigi Vanvitelli”, Via Vivaldi 43, 81100 Caserta, Italy; giovanna.cimmino@unicampania.it (G.C.); claudia.gravina@unicampania.it (C.G.); severina.pacifico@unicampania.it (S.P.); 2 Department of Chemical Sciences, University of Napoli Federico II, Via Cinthia 4, 80126 Napoli, Italy; silvana.pedatella@unina.it; 3 Department of Sciences, University of Basilicata, Via dell’Ateneo Lucano 10, 85100 Potenza, Italy; mauro.denisco@unibas.it

**Keywords:** selenosugars, hydro-cinnamic acids, UHPLC–HRMS, anti-radical activity, cytotoxicity

## Abstract

Selenosugars are gaining growing interest due to their antioxidant efficacy, and their ability to inhibit glycosidases, repair skin tissue or reduce endothelial dysfunction. Among selenosugars, those in which selenium replaces heterocyclic oxygen in a 5-membered sugar were our focus, and their coupling with phenolic compounds appears to be a strategy aimed at producing new compounds with enhanced antioxidant efficacy. In this context, the Mitsunobu reaction has been advantageously explored to obtain *trans*-*p*-coumaroyl-1,4-deoxy-2,3-*O*-isopropylidene-4-seleno-d-ribose, *trans*-caffeoyl-1,4-deoxy-2,3-*O*-isopropylidene-4-seleno-d-ribose, and *trans*-feruloyl-1,4-deoxy-2,3-*O*-isopropylidene-4-seleno-d-ribose. These compounds underwent removal of the iso-propylidene group, to provide the corresponding hydroxycinnamoyl-1,4-deoxy-4-seleno-d-ribose. All compounds were characterized by Nuclear Magnetic Resonance (NMR) spectroscopy and High-Resolution Mass Spectrometry (HRMS). This latter technique was pivotal for ensuing cellular metabolomics analyses. In fact, after evaluating the anti-radical efficacy through 2,2-diphenyl-1-picrylhydrazyl (DPPH) and 2,2′-azino-bis(3-ethylbenzothiazoline-6-sulfonic acid) (ABTS) methods, which underline the massive role of the phenolic moiety in establishing efficacy, the compounds, whose cytotoxicity was first screened in two highly oxidative-stress-sensitive cells, were tested for their wound healing properties towards human HaCaT keratinocytes cells. Caffeoyl- and feruloyl selenosugars exerted a dose-dependent repair activity, while, as highlighted by the metabolomic approach, they were poorly taken up within the cells.

## 1. Introduction

The cosmeceutical industry is in constant evolution, driven by the increasing demand for products that combine cosmetic and pharmaceutical aspects to promote skin health and beauty [1]. It has been estimated that the related global market will increase by 2032 with a compound annual growth rate (CAGR) of 9.1% [2]. Indeed, the constant search for new innovative molecules represents a fundamental pillar in promoting skin health and well-being [3]. Simultaneously, it is essential to recognize that our body undergoes metabolic processes and environmental factors that generate reactive oxygen or nitrogen species, leading to a condition known as oxidative stress [4]. This phenomenon has been closely associated with the pathogenesis of several human diseases, including skin aging [5,6]. In this context, the benefits of using natural derivatives of cinnamic acid to improve the state and appearance of skin and hair have long been known [7]. In particular, hydroxycinnamic acids (HCAs) have garnered significant attention due to their widespread presence in various plant-derived foods, particularly in fruits, vegetables, and seeds [8]. From a phytochemical point of view, HCAs are biosynthetically derived from the shikimate pathway and are precursors of a number of polyphenols [9]. Common HCAs found in nature include *p*-coumaric, caffeic, ferulic, and sinapic acids, sharing a C6-C3 structure, and differing in the number and location of hydroxyl groups and other substituents on the aromatic ring. HCAs are seldom found free; instead, they are frequently esterified with tartaric and quinic acids, or conjugated to different carbohydrates. Apart from being able to neutralize the harmful effects of free radical species, they have also been recognized for their anti-tyrosinase and anti-collagenase properties and UltraViolet (UV) ray protection. In this scenario, they have been suggested as anti-aging, anti-inflammatory, and anti-hyperpigmentation agents [10].

Herein, in the pursuit of searching for and improving innovative molecules for cosmeceutical applications, three HCAs (caffeic, ferulic, and *p*-coumaric acid) were chemically linked to an Se-sugar-type structure via Mitsunobu reaction, hypothesizing that the potential bioactivity of these compounds could benefit not only the HCA moiety but also the selenium atom integrated into the sugar ring. Although previous research has focused on the active role of selenosugars as possible antioxidants, glycosidase inhibitors (strong targets for the development of antitype 2 diabetes, anticancer, and antiviral agents), skin repair tissue, and reducing endothelial dysfunction [11,12,13,14,15], as far as the authors are aware, this is the first study on Se-sugars linked to HCAs in biological systems. After evaluating potential cell cytotoxic effects, the biological investigation was driven to assessment of their radical scavenging and wound-healing properties. This latter has been described as a process, complex but essential, related to the restoration of tissue integrity and functionality, which protects from infections and other external threats [16]. Indeed, human in vitro models are highly desirable to enhance the clinical applicability of basic research findings and to reduce animal testing in line with the “3Rs” principle (Replacement, Reduction, and Refinement). Thus, various in vitro approaches have been developed to model human skin, ranging from simple 2D monocultures of key skin cells to complex 3D tissue models (e.g., reconstructed human epidermis) [17]. However, single-cell-type 2D models proved to be reliable in studying the response to injury and stress after treatment with molecules able to enhance cell migration and re-epithelialization [18].

The results of biological assays have been compared to those acquired for their unconjugated counterparts. Moreover, the study aimed to obtain a picture of their internalization in HaCaT keratinocyte cell line through Ultra High Performance Liquid Chromatography–Quadrupole Time Of Flight–Mass Spectrometry (UHPLC–QqTOF–MS) metabolomic tools.

## 2. Materials and Methods

### 2.1. Preparation of Se-Glycoconjugates and Structure Elucidation

The Fourier Transform NMR Varian 500 Unity Inova spectrometer is applied to record the ^1^H- and ^13^C NMR spectra at 500 and 125 MHz, respectively, or 400 and 100 MHz, respectively, when a Bruker DRX 400 MHz spectrometer (Bruker, Milan, Italy) is used. Unless otherwise noted, CDCl_3_ is employed as the solvent. ^1^H–^1^H COSY experiments provided evidence for the proton couplings. The HSQC and HMBC pulse sequences determined the heteronuclear chemical shift correlations [19].

ESI–QqTOF HR–MS and MS/MS data were acquired in the negative ESI mode using the AB SCIEX Triple TOF^®^ 4600 mass spectrometer (AB Sciex, Concord, ON, Canada) equipped with a DuoSpray^TM^ ion source. The Calibrant Delivery System (CDS) automatically injected the calibrant solution through the APCI probe before MS and MS/MS experiments. The source parameters were the following: curtain gas 35 psi, nebulizer and heated gases 60 psi, ion spray voltage −4.5 kV, interface heater temperature 600 °C, declustering potential (DP) −70 V and collision energy (CE) −35 ± 15 V. The instrument was controlled by Analyst^®^ TF 1.7 software (AB Sciex, Concord, ON, Canada), whereas MS data were processed by PeakView^®^ 2.2 software (AB Sciex, Concord, ON, Canada).

Se-Glycoconjugates containing caffeic and ferulic acids were synthesized following the methodology previously outlined by Serpico et al. [19]. Additionally, novel molecules with *p*-coumaric acid were synthesized using the same approach. The primary hydroxyl of Se-sugar and hydroxycinnamic acid (ferulic, *p*-coumaric and caffeic acid) were employed in the Mitsunobu reaction to synthesize the corresponding Se-Glycoconjugates. NMR spectroscopy was used to characterize the synthesized compounds.

#### 2.1.1. Mitsunobu Reaction: Preparation of Glycoconjugates. Typical Procedure

Diisopropyl azodicarboxylate (DIAD) (0.303 mL, 1.5 mmol) was added to a magnetically stirred solution of triphenylphosphine (TPP) (0.393 g, 1.5 mmol) in anhydrous THF (2.3 mL) at 0 °C under N_2_. After 15 min, a solution containing seleno derivative **2** (0.200 g, 1.0 mmol) and *p*-coumaric acid (0.246 g, 1.5 mmol) in anhydrous THF (3.0 mL) was added dropwise. The reaction mixture was stirred at room temperature for three days. The solvent was evaporated under reduced pressure and replaced with AcOEt. The organic layer was washed with brine and dried (Na_2_SO_4_). The evaporation of the solvent under reduced pressure gave a crude residue that was purified by silica gel column chromatography (hexane-diethyl ether 7:3 (*v*/*v*)) to give the pure product **3a** as a pale yellow-syrup (0.280 g, 0.73 mmol, 73%). ^1^H NMR (500 MHz): δ 1.35 (s, 3H, CH_3_), 1.55 (s, 3H, CH_3_), 3.06 (m, 1H, H1a), 3.28 (dd, J_1b-1a_ = 12.0 Hz, J_1b-2_ = 4.6 Hz, 1H, H1b), 3.79 (m, 1H, H4), 4.29 (dd, J_5a-5b_ = 11.5 Hz, J_5a-4_ = 8.9 Hz, 1H, H5a), 4.40 (dd, J_5b-5a_ = 11.6 Hz, J_5b-4_ = 5.8 Hz, 1H, H5b), 4.79 (dd, J_3-2_ = 5.4 Hz, J_3-4_ = 1.8 Hz, 1H, H3), 5.04 (m, 1H, H2), 6.31 (d, J_7-8_ = 16.0 Hz, 1H, H7), 6.87 (d, J_11-10_ = 8.4 Hz, 2H, H11), 7.44 (d, J_10-11_ = 8.4 Hz, 2H, H10), 7.66 (d, J_8-7_ = 16.0 Hz, 1H, H8). ^13^C NMR (125 MHz): δ 167.1, 158.0, 145.3, 130.1, 127.0, 115.9, 114.8, 110.8, 87.3, 85.0, 65.6, 46.7, 29.7, 26.7, 24.7. Anal. Calcd for C_17_H_20_O_5_Se: C, 53.27; H, 5.26; Se, 20.60. Found: C, 53.31; H, 5.16; Se, 20.65.

#### 2.1.2. Removal of *O*-isopropylidene. Typical Procedure

A solution (3.3 mL) of CH_3_COOH/H_2_O (8:2, *v*/*v*) was added to compound **3a** (0.383 g, 1.0 mmol). The mixture was stirred at 80 °C for 2 h. Then, the solvent was evaporated under reduced pressure; next, the organic layer was washed with diethyl ether. The crude residue was purified by silica gel column chromatography (chloroform) to afford compound **3b** as an amorphous solid (0.240 g, 0.70 mmol, 70%). ^1^H NMR (500 MHz, CD_3_OD): δ 2.84 (dd, J_1a-1b_ = 10.1 Hz, J_1a-2_ = 5.3 Hz, 1H, H1a), 3.06 (dd, J_1b-1a_ = 10.1 Hz, J_1b-2_ = 5.0 Hz, 1H, H1b), 3.71 (m, 1H, H4), 4.05 (dd, J_3-4_ = 6.1 Hz, J_3-2_ = 3.2 Hz, 1H, H3), 4.24 (dd, J_5a-5b_ = 11.3 Hz, J_5a-4_ = 7.4 Hz, 1H, H5a), 4.37 (td, J_2-1a_ = 5.3 Hz, J_2-1b_ = 5.0 Hz, J_2-3_ = 3.2 Hz, 1H, H2), 4.56 (dd, J_5b-5a_ = 11.3 Hz, J_5b-4_ = 6.6 Hz,1H, H5b), 6.35 (d, J_7-8_ = 15.9 Hz, 1H, H7), 6.82 (d, J_11-10_ = 8.5 Hz, 2H, H11), 7.48 (d, J_10-11_ = 8.5 Hz, 2H, H10), 7.65 (d, J_8-7_ = 15.9 Hz, 1H, H8). ^13^C NMR (125 MHz, CD_3_OD): δ 167.4, 160.0, 145.5, 129.8, 115.4, 113.4, 78.1, 75.7, 66.6, 41.1, 23.4. Anal. Calcd for C_14_H_16_O_5_Se: C, 48.99; H, 4.70; Se, 23.00. Found: C, 49.05; H, 4.66; Se, 22,97.

### 2.2. Anti-Radical Capacity Assessment

The ability of the synthesized compounds to scavenge free radicals was evaluated by 2,2-diphenyl-1-picrylhydrazyl (DPPH) and 2,2′-azino-bis(3-ethylbenzothiazoline-6-sulfonic acid) (ABTS) tests, performed as previously reported [20]. In both cases, the hydroxycinnamoyl Se-glycoconjugates were tested at 5, 12.5, 25, and 50 μM final concentrations and the responses were expressed compared to those recorded for the corresponding bare hydroxycinnamic acids.

### 2.3. Cell Culture and Cell-Based Experiments

Human keratinocyte (HaCaT) and human neuroblastoma (SH-SY5Y) cell lines were cultured in Dulbecco’s Modified Eagle’s Medium (DMEM) supplemented with 10% fetal bovine serum, 50.0 U/mL of penicillin and 100.0 µg/mL of streptomycin, at 37 °C in a humidified atmosphere containing 5% CO_2_.

#### 2.3.1. MTT Cell Viability Assay

Cells were seeded in 96-multiwell plates at a density of 1.0 × 10^4^ cells/well. After 24 h, cells were exposed to both Se-glycoconjugates and bare hydroxycinnamic acids, tested at 5, 10, 50, 100, and 250 µM (final concentration levels). After 24 h of incubation, the MTT assay was performed. For this purpose, MTT (3-(4,5-dimethyl-2-thiazolyl)-2,5-diphenyl-2H-tetrazolium; 0.5 mg/mL) was added to the FBS-free culture medium and allowed to stand for 4 h at 37 °C in a 5% CO_2_ humidified atmosphere. After removing the MTT solution, the resulting formazan dye was dissolved using 100 µL of DMSO. Subsequently, the absorbance at 570 nm for each well was measured using a Victor3 Perkin Elmer absorbance reader (Perkin Elmer/Wallac, Waltham, MA, USA). The cell mitochondrial redox activity inhibition (RAI, %) was calculated, compared with the untreated control cells, applying the following formula [21]:

RAI (%) = ((Abs untreated cells) − (Abs treated cells)/(Abs untreated cells)) × 100

#### 2.3.2. Wound Healing Assay

HaCaT cells were seeded in 60-mm plates (5 × 10^5^ cells/plate). Once the cell monolayer appeared confluent, a wound was manually caused by scraping the cell layer with a sterile p200 micropipette tip, followed by three washing steps with Dulbecco’s Phosphate-Buffered Saline (DPBS; 1 mL each). Subsequently, the cells were exposed to the investigated compounds (5, 10, 50, and 100 µM), whereas a negative control was assessed by adding only the culture medium. The wound size was measured at different times: 0, 3, 6, 24, 48, and 72 h, as reported in Gravina et al. [22].

### 2.4. UHPLC–HRMS Cell Metabolomic Target Analysis

**Figure 1:**
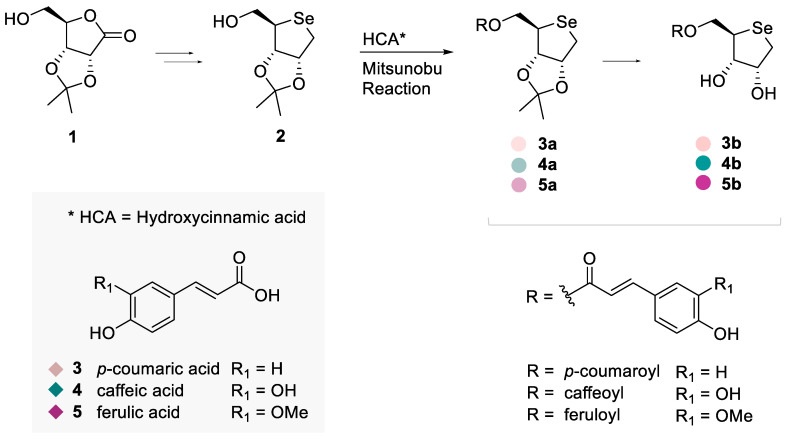
Synthetic pathway of Se-glycoconjugates (**3a**, **4a**, **5a**, **3b**, **4b**, **5b**) and their chemical structures and unconjugated hydroxycinnamic acids (**3**, **4**, **5**).

HaCaT cells (1.5 × 10^6^ cells in a Petri dish) were treated with Se-glycoconjugates (100 µM, final concentration), whose chemical structures are depicted in Figure 1. After 24 h exposure time, the culture medium was removed, and the cells were immediately quenched with 1 mL of ice-cold MeOH to stop metabolic activity. Then, the cells were scraped and collected in Eppendorf tubes, and centrifuged at maximum rpm for 10 min. After removing the supernatants, the obtained pellets were extracted three times using 0.5 mL of a cold MeOH:H_2_O (4:1, *v:v*) solution. The resulting extracts underwent UHPLC–HRMS analysis [23].

To this purpose, the NEXERA UHPLC system (Shimadzu, Tokyo, Japan) was used, equipped with a Luna^®^ Omega C18 column (1.6 μm particle size, 50 × 2.1 mm i.d., Phenomenex, Torrance, CA, USA). The elution was isocratic, employing a binary solution made of 75% water and 25% acetonitrile (both acidified with 0.1% formic acid). The flow rate was set at 500 μL/min (in a total run time of 5 min) and the injection volume was 2 μL. The UHPLC was connected in series to the AB SCIEX Triple TOF^®^ 4600 mass spectrometer, operating with the same parameters previously used for the structure elucidation. Quantitation was achieved by using calibration curves built up for each compound in the concentration range 25–200 µM (**3a**, **4a**, **5a**) or 2–100 µM (**3a**, **4a**, **5a**), corresponding to the peculiar linearity range for the two series of synthesized compounds.

### 2.5. Statistical Analysis

Results from anti-radical assays were based on two independent experiments, each performed in triplicate (in total, 2 × 3 measurements). For MTT assay, six replicates from two independent experiments (in total: 2 × 6 measurements) were considered whereas, finally, the wound closure (%) was calculated from measurements of two independent experiments performed in triplicate (in total: 2 × 3 measurements). All data were expressed as mean values ± standard deviation (SD). *p* < 0.05 values indicated a statistically significant difference. Moreover, data from anti-radical tests underwent multivariate analysis–Hierarchical Clustering, by applying the group average cluster method and Euclidean distance type (OriginPro 2015 software; OriginLab Corp., Northampton, MA, USA).

## 3. Results and Discussion

This study aims to evaluate the efficacy of hydroxycinnamoyl Se-glycoconjugates as novel cosmeceutical ingredients, capable of exerting anti-radical effects and wound-healing properties, which could define their role in skin health and well-being. Herein, the already reported library of synthesized compounds [19] was enriched by two derivatives of *p*-coumaric acid. This has been extensively studied either in free or in conjugated form for several biological properties, ranging from anti-atherosclerosis to antioxidant, anti-inflammatory, and anti-cancer properties [24,25]. Its application as an anti-melanogenic agent in the cosmetic sector has also been recently reviewed [26].

Furthermore, a targeted cell metabolomic approach, carried out by UHPLC–HRMS tools, defined the uptake levels in HaCaT human keratinocyte cell line.

### 3.1. Synthesis and Characterization of Hydroxycinnamoyl Se-Glycoconjugates

In Figure 1, the synthetic pathway of Se-glycoconjugates has been reported. The synthesis of organo-Se compounds **4a**, **4b**, **5a**, and **5b** faithfully followed the methodology outlined by Serpico et al. [19], employing the same Se-sugar, covalently bonded to caffeic (**4**) and ferulic (**5**) acid residues, respectively. In brief, the strategy firstly involved the synthesis of the d-seleno-deoxy-sugar **2** from the d-ribonolactone (**1**). Subsequently, employing the primary hydroxyl group of the hydroxycinnamic acids (HCAs) **4** and **5**, Se-glycoconjugates were synthesized via the Mitsunobu reaction, as previously detailed [19]. The introduction of *p*-coumaric acid (**3**) in novel compounds **3a** and **3b** was achieved by implementing Serpico’s synthesis. Mono- and bi-dimensional NMR data confirmed all the structures (Figures S1 and S2).

**Figure 2:**
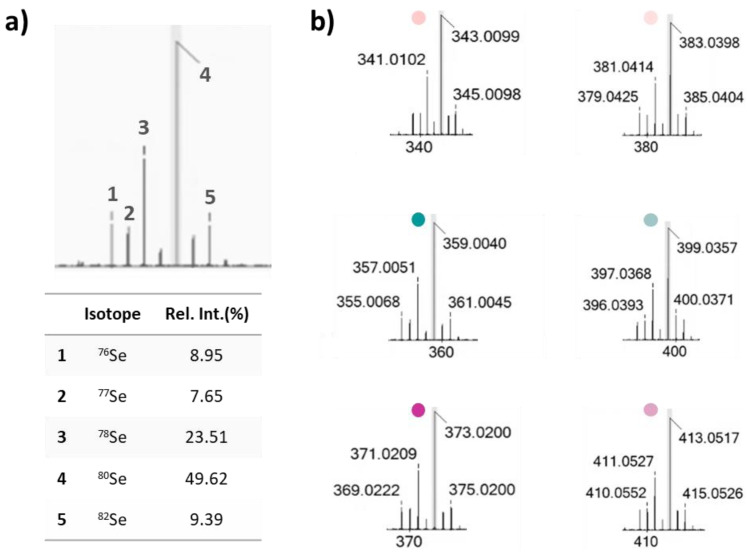
(**a**) Selenium isotopic pattern; (**b**) deprotonated ions of compounds **3a**, as detected in TOF–MS spectra. The compound labeling is referred to in Figure 1.

Moreover, a high-resolution tandem mass spectrometry systematic study, herein performed for the first time on all the Se-glycoconjugates, made their identification efficient, straightforward, and unambiguous, being pivotal to the cell metabolomic approach, carried out by UHPLC–HRMS tools. In Figure 2, TOF–MS and MS/MS spectra are reported, together with the fragmentation pathways that represent the “identity card” of each molecule [23].

When analyzed in negative ion mode, all the investigated compounds exhibited deprotonated molecular ions, taking advantage of the occurrence of almost one acidic proton on the HCA aromatic moiety. The peculiar selenium isotopic pattern allowed us to recognize at a glance the presence of this element in the TOF–MS spectra (Figure 2). In fact, the intensity ratios between the deprotonated ions containing ^80^Se and ^78^Se, ranging from 2.08 to 2.18 (experimental values), reflected those ascribed to stable Se isotopes (49.62%/23.51%) [27].

**Figure 3:**
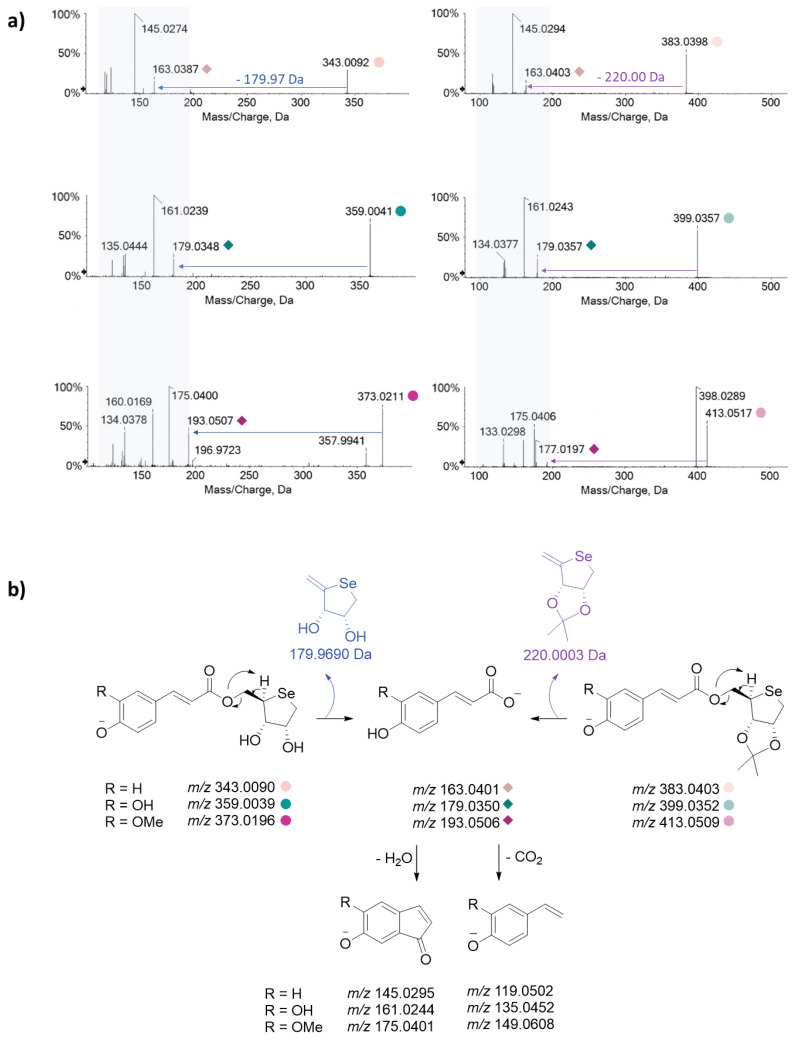
(**a**) TOF–MS/MS spectra of Se-glycoconjugates under investigation, and (**b**) the hypothesized fragmentation pathways (*m/z* theoretical values are reported below each structure). The compound labeling is referred to in Figure 1.

The most intense ions (containing ^80^Se) underwent collision-induced dissociations. The main fragmentation routes saw the loss of the selenosugar as a dehydrated species of 220.0003 or 179.9690 Da (exact mass values), depending on whether or not the sugar hydroxyl groups at C-2 and C-3 were protected by the iso-propylidene function (Figure 3). The resulting fragment ions at *m/z* 163.04, 179.03, and 193.05 corresponded to the free deprotonated HCAs (*p*-coumaric, caffeic, and ferulic acids, respectively), whose MS fragmentation mainly involved dehydration and decarboxylation reactions.

### 3.2. Anti-Radical Capacity in Cell-Free Assays

**Figure 4:**
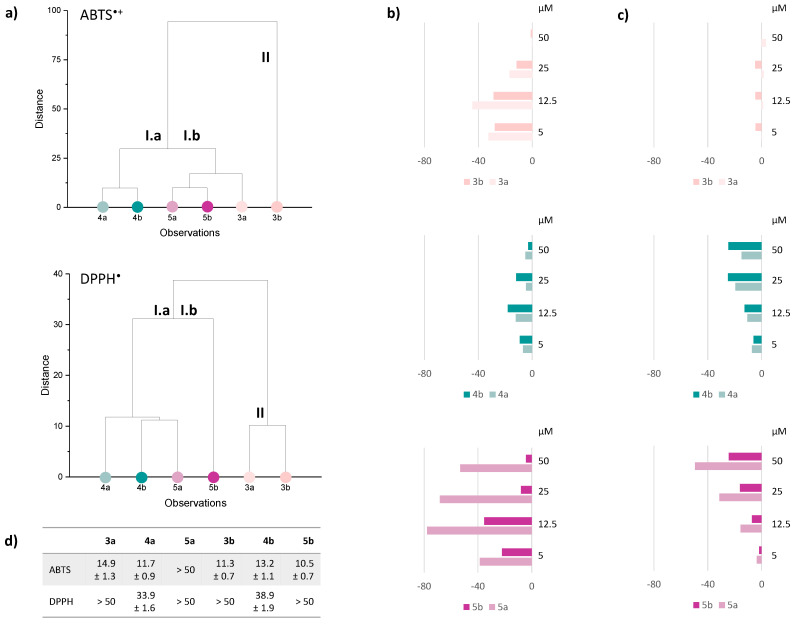
(**a**) Hierarchical Cluster Analysis, based on anti-radical tests. The radical scavenging capacity (RSC, %) of Se-glycoconjugates was also expressed as the decrease vs. that recorded for unconjugated hydroxycinnamic acids towards (**b**) ABTS^•+^ and (**c**) DPPH^•^. (**d**) IC_50_ values (µM) in ABTS and DPPH tests.

The bioactivity of a compound is closely related to its chemical features, with structural modifications significantly impacting its biological properties, including radical-scavenging capabilities [28]. With this awareness, Se-glycoconjugates (**3a**, **3b**, **4a**, **4b**, **5a**, **5b**) were tested for their capacity to scavenge DPPH^•^ and ABTS^•+^ probes, and the results were compared to pure HCAs (**3**, **4**, **5**). Hierarchical cluster analysis was applied to investigate the dissimilarity of data related to the anti-radical capacity of Se-glycoconjugates with respect to the corresponding HCAs towards the radical probe employed. The obtained dendrograms highlighted two distinct clustering patterns (Figure 4a). In the ABTS assay, they had a significant distance value of 94%. Specifically, cluster I comprised two subgroups (Ia and Ib), with caffeic acid glycoconjugates **4a** and **4b** on one side and ferulic acid derivatives **5a** and **5b** together with **3a** on the other. Only **3b** was part of cluster II. Concerning the DPPH test, the maximum distance reached was 39%, but a more pronounced segregation based on the HCA basic skeleton was observed. Indeed, regardless of the iso-propylidene function on the sugar moiety, *p*-coumaric acid derivatives showed the same capacity to neutralize the DPPH radical compared to the bare HCA (cluster II).

Based on clustering, anti-radical efficacy data were plotted as the decrease in radical scavenging capacity (RSC, %) vs. ABTS^•+^ and DPPH^•^ compared to the corresponding HCAs (Figure 4, panels b and c, respectively). As a general trend, the response in the ABTS test was considerably higher than that recorded against DPPH radical, especially for those compounds bearing *p*-coumaric (**3a**, **3b**) and ferulic acid (**5b**), as shown by IC_50_ values (Figure 4d). The RSC % of caffeic acid Se-glycoconjugates (**4a**, **4b**) towards both radical probes was comparable at all the tested doses and showed only a slight difference with respect to the bare HCA, having IC_50_ values of 9 µM and 30.6 µM vs. ABTS^•+^ and DPPH^•^, respectively. According to the literature, the glycosylation usually hampers the Single-Electron Transfer/Hydrogen-Atom Transfer (SET/HAT) mechanism in the DPPH assay, since the hydroxyl groups and the conjugated structure are less available due to a greater steric hindrance. In the case of compounds **4a** and **4b,** the catechol moiety preserves the activity, as the *ortho* OH group can stabilize the phenoxy radical via electron donation [29], contributing to the formation of stable semiquinone radicals [30]. Additionally, the electron-donating characteristics of the methoxy group stabilized the phenoxy radical generated during the reaction [31]. Notably, differences from this activity order were observed with ABTS^•+^ assay, where an increase in the number of hydroxyl groups in the aromatic ring did not consistently correlate with an improvement in radical scavenging capacity [32]. Specifically, Se-glycoconjugates of *p*-coumaric acid (**3a** and **3b**) demonstrated inactivity in the DPPH assay but exhibited antioxidant capacity against ABTS^•+^, even surpassing the activity of ferulic acid Se-glycoconjugate **5a**.

### 3.3. Cytotoxicity Evaluation

A first assessment of the potential cytotoxic effects ascribable to the Se-glycoconjugates under study is mandatory to investigate their putative applications in the cosmeceutical sector. To this aim, the MTT test was performed on the HaCaT keratinocyte cell line, a well-established in vitro model for skin-related interventions, as they represent the predominant cell type in the epidermis, acting as its first barrier against external harmful stimuli [33]. In addition, the molecules were also tested on the SH-SY5Y cell line, undifferentiated cells sensitive to oxidative stress, presenting an epithelial-like phenotype.

**Figure 5:**
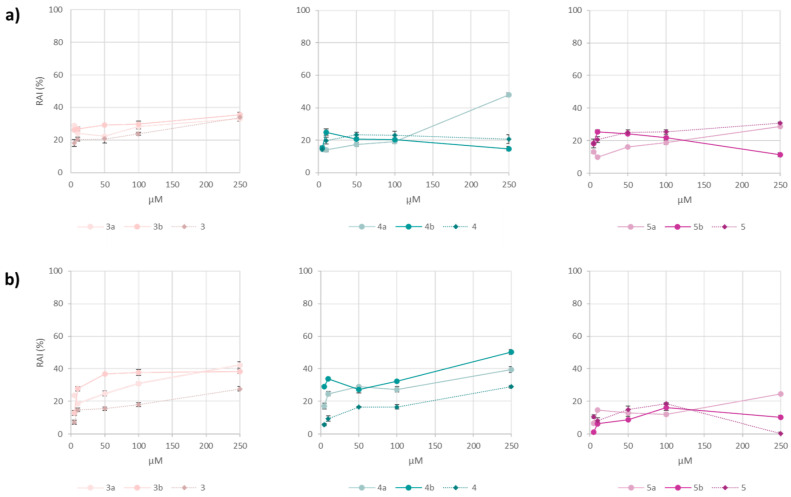
Redox activity inhibition (RAI%) of Se-glycoconjugates and their unconjugated hydroxycinnamic acids towards (**a**) HaCaT and (**b**) SH-SY5Y cell lines.

The preliminary cytotoxicity screening of Se-glycoconjugates was performed at concentrations ranging from 5 to 250 μM (Figure 5). The general trend showed a slightly higher redox activity inhibition than the corresponding bare HCAs, tested for comparison. However, mild cytotoxic effects on SH-SY5Y cell line were observed only at the highest tested dose, except for compound **4b**, responsible for the 50% inhibition, and **4a**, which was able to compromise the keratinocyte redox activity for 48%. On the contrary, when ferulic acid represented the hydroxycinnamoyl moiety (**5a** and **5b**), the compounds did not influence the redox status of both cell lines at any of the tested concentrations.

These results highlight the feasibility of safely using these compounds in the cosmeceutical sector up to the 100 µM concentration and lay the groundwork for further investigations, such as into their capability to modulate cell migration in tissue repair.

### 3.4. Wound Healing Properties on HaCaT Cell Line

**Figure 6:**
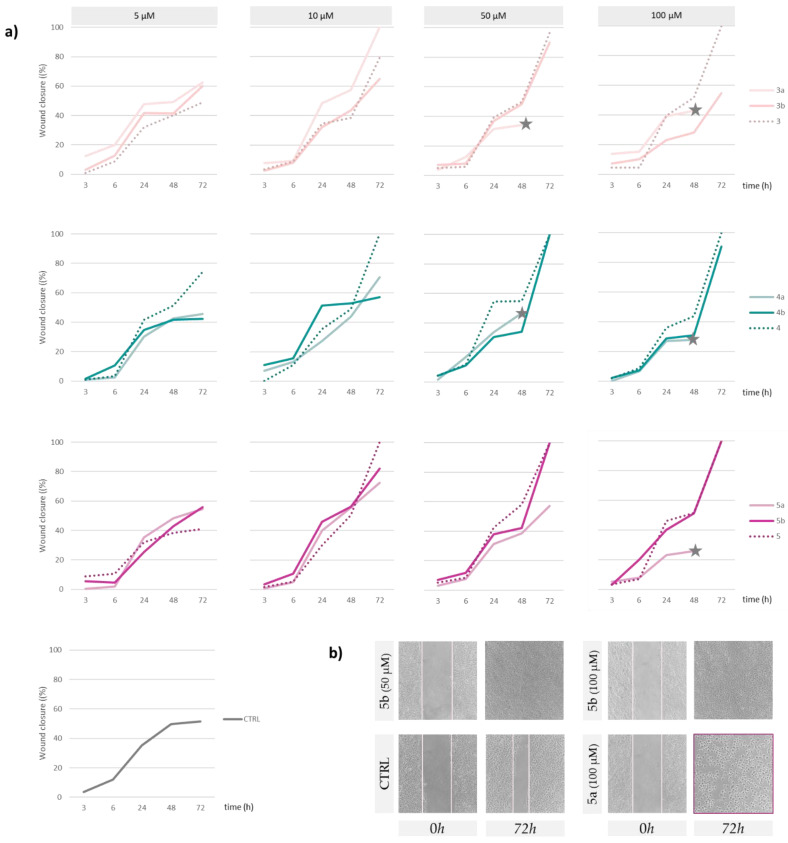
Scratch wound assay, carried out on HaCaT cells. (**a**) Wound closure (%) from measurements taken at different times (3, 6, 24, 48, and 72 h) after treatment with Se-glycoconjugates under study and unconjugated hydroxycinnamic acids (5, 10, 50, and 100 µM). (**b**) Representative images of untreated cells (CTRL) and those treated with compounds **5a** and **5b** at t_0_ and after 72 h. Images were acquired by Inverted Phase Contrast Brightfield Zeiss Primo Vert Microscope.

The search for innovative approaches to enhance the wound healing process leveraging the well-established regenerative properties of HCAs and novel Se-sugars was the driving force in exploring the effects of the synthesized compounds on HaCaT cell line [34]. Indeed, keratinocytes play a central role in the repair of cutaneous injuries [35], as they form the outer layer of the skin and serve a crucial function in the reconstruction of damaged tissues [36] through cell proliferation and migration, and the production of growth factors [37,38]. The impact of synthesized Se-glycoconjugates and unconjugated HCAs was evaluated by using an in vitro scratch wound model applied to HaCaT cell line. Briefly, a simulated wound was created by scraping the confluent keratinocyte cell layer (“scratch wounding”). This process causes cellular trauma, triggering a series of events, including cell proliferation, protein synthesis, and alterations in cell viability, migration, gene expression, and differentiation, ultimately leading to the opening of a wound area that needs to be covered and closed [16]. The healing process was assessed by measuring wound width at various time intervals (0, 3, 6, 24, 48, 72 h), and calculating the wound closure as a percentage compared to t_0_ (Figure 6a) after treatment at four concentration levels (5, 10, 50 and 100 µM). Results obtained after treatment with the lowest dose were comparable to the control, reaching a maximum of 60% only in the case of *p*-coumaric acid derivatives **3a** and **3b**. However, HaCat cells appeared sensitive to Se-glycoconjugates **3a**, **4a**, and **5a**, and cell suffering was detected after 48 h of observation. In fact, cell morphology changed, cells shrunk and lost adhesion, suggesting a potential cytotoxicity due to prolonged exposure (Figure 6b). This could be due to the presence of the iso-propylidene moiety and underlined the importance of considering the potential impact of protective groups used in carbohydrate synthesis on cellular behavior in the development of glycoconjugates for biological applications. On the contrary, the repair capacity of Se-glycoconjugates **4b,** and **5b**, enhanced by increasing the compound concentration, proved to be similar to the corresponding HCA even at lower doses.

It has been previously reported that HCAs play a crucial role in stimulating skin cell proliferation, besides promoting collagen synthesis and exhibiting antimicrobial activity [7,39,40,41,42]. These attributes not only suggest their efficacy in maintaining skin health under normal conditions but also their potential in treating several skin disorders (e.g., acne, dermatitis, eczema, psoriasis). Among HCAs, ferulic acid was involved in safeguarding essential skin components, including keratinocytes, fibroblasts, collagen, and elastin, regulating melanin production, promoting angiogenesis, and accelerating wound healing. Consequently, it has gained wide application in skincare formulations [43]. Moreover, its positive effects in mitigating inflammation, initiating antioxidant pathways, and fostering the formation of new skin tissue have been underlined [44]. Staniforth et al. [45] demonstrated that ferulic acid effectively inhibits metalloproteinase-2 (MMP-2) and metalloproteinase-9 (MMP-9) activation, playing a crucial role in preventing photo-saturation and initiating processes associated with photo-carcinogenesis induced by UVB radiation. Furthermore, Lin et al. [46] discussed its capability to increase hypoxia-induced HIF-1, triggering hypoxia-responsive reactions and elevating the production of vascular endothelial growth factor (VEGF) and platelet-derived growth factor (PDGF). Caffeic acid’s potential wound-healing effects were explored in a study involving skin-incised mice, revealing impacts on anti-inflammatory activity and the wound-healing processes, affecting myeloperoxidase activity, lipid peroxidation, phospholipase A_2_ activity, and collagen-like polymer synthesis [47]. Its phenethyl ester has been shown to accelerate cutaneous cicatrization in rat models, by reducing oxidative stress and enhancing blood flow through nitric oxide-mediated vasorelaxation [48]. Moreover, research illustrates that the incorporation of *p*-coumaric acid into bio-polymeric scaffolds enhances the cell proliferation of diabetic wounds, impacting the production of MMP-9 and transforming growth factor-beta 3 (TGF-β3) [49].

On the other hand, as regards the potential roles of Se-sugars in this context, as previously mentioned, Davies and Schiesser [12] explored the promising potential of water-soluble selenium-containing carbohydrate derivatives, such as 1,4-anhydro-4-seleno-d-talitol (SeTal), in facilitating the healing of injured skin tissue. This sugar has intriguing antioxidant and skin-healing qualities. In fact, it has been demonstrated that the topical treatment with SeTal reduced scratching behavior in mice, cutaneous severity ratings, and inflammatory markers caused by 2,4-dinitrochlorobenzene-induced cutaneous lesions, similar to atopic dermatitis [50]. These findings were also promoted by gelatine and alginate polymeric films enriched with SeTal [51]. Moreover, the pH-sensitive release of antioxidant Se-glycoconjugates through a flexible polymeric patch is a recent development in the field of wound healing [34].

### 3.5. Compound Uptake Evaluation in HaCaT Cells: A UHPLC–HRMS Target Analysis

A metabolomic approach was carried out to estimate the uptake of the Se-glycoconjugates under study in HaCaT cells in the first 24 h of treatment with a 100 µM dose, which ensured the absence of basal cytotoxicity from MTT data [52], neither morphological changes (e.g., cell shrinkage) nor cellular damage (e.g., cell detachment). To the best of our knowledge, this is the first work reporting on the internalization of Se-glycoconjugates by human keratinocytes. The enrolment in the experimental setting of the molecules of the series “a” and “b” was designed to correlate the internalization with the different ability to counteract the healing process. The UHPLC–HRMS analysis also offered the opportunity to evaluate the putative structural changes of the target compounds due to metabolic processes within cells.

**Figure 7:**
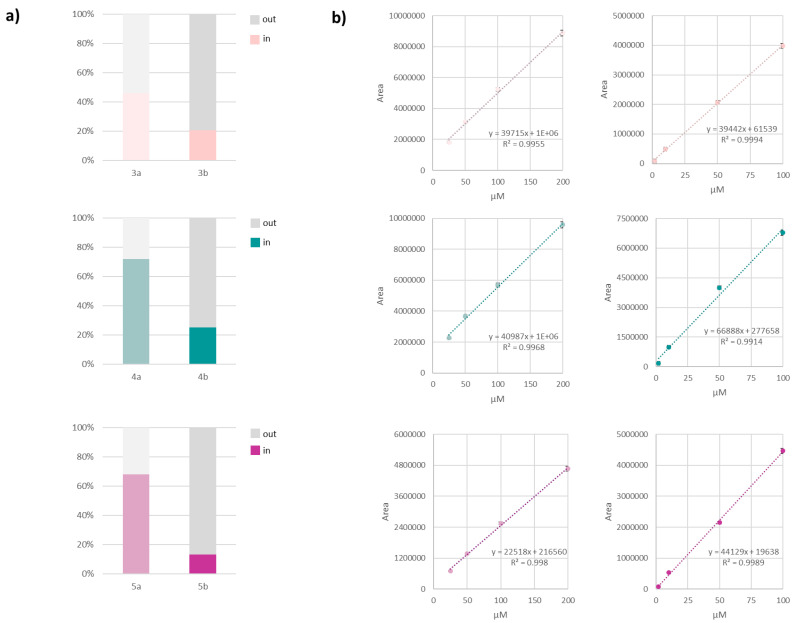
(**a**) Results from cell metabolomic target analysis, and (**b**) the employed calibration curves for each Se-glycoconjugate.

At the end of the planned exposure time, the culture medium, likely containing the not internalized compounds, was removed and the cells were quenched, scraped, and properly extracted to maximize the compound recovery from the lysate cell pellet. The obtained extracts underwent a first untargeted UHPLC–HRMS analysis to check their presence and structural integrity. The overview of the total ion current chromatograms did not highlight signals related to changes in the molecule identity. Indeed, apart from the investigated Se-glycoconjugates, other peaks showing the presence of selenium were not detected, and their TOF–MS and MS/MS were superimposable to those recorded for pure molecules. Then, calibration curves were constructed, and a single ion monitoring target analysis was performed to estimate the cellular uptake, herein reported as % values (Figure 7).

Cell metabolomic results highlighted a different capability to penetrate cellular membranes. Se-glycoconjugates belonging to the “a” series (maintaining the iso-propylidene sugar protecting group) were able to penetrate the cells more easily, likely due to overall amphiphilicity higher than the counterpart with the free Se-sugar moiety, regardless of the structure of the hydroxycinnamic acid. Compound **4a**, bearing caffeic acid as the HCA moiety, was the most prone to internalization, reaching 72%. Compound **5a** showed a slightly lower ability (68%), whereas **3a** was 1.6-fold less capable of crossing the membrane lipid layer. The results obtained for the “b” series molecules confirmed that the presence of caffeic acid was useful in targeting the compound inside the cell but in this case the order for *p*-coumaric and ferulic acid derivatives was inverted, with this latter accounting only for 13%. This suggests that different interactions with the cell membrane constituents drive the molecule uptake, laying the basis for further investigation, as the mechanisms could be multifaceted and hard to disclose considering that, on the membrane surface, hundreds of specific lipid–lipid, lipid-protein, and protein–protein interactions occur [53]. Indeed, it has been demonstrated that the interaction and penetration of small organic acids through biological membranes are influenced by substituents in their main structure [54], so much so that compounds with structural similarity but different functional groups, affecting lipophilicity, interact differently with membranes. Ferulic acid was observed to exert a more pronounced influence on the lipid bilayer structure, suggesting a greater ability to cross membranes [55]. It also showed a higher percutaneous absorption compared to caffeic acid [56]. However, this theory is valuable when considering bare HCAs, whereas their conjugation could lead to different and unexpected responses that take into consideration other issues, related not only to the compound structural features (e.g., size, shape, packing efficiency, polarity, capacity to establish weak intermolecular interactions), but also to the membrane’s biophysical properties (integrity, lipid order, hydration, fluidity, redox potential) [53].

## 4. Conclusions

A successful synthetic route resulted in the preparation of novel *p*-coumaric acid Se-glycoconjugates, which were thoroughly characterized using spectroscopic and spectrometric techniques. When tested in cell-free assays against ABTS^•+^ and DPPH^•^ together with other organo-Se-compounds bearing caffeic or ferulic acid, the radical scavenging capacity variations were attributed to the specific hydroxycinnamoyl moiety and type of radical probe. A preliminary cytotoxicity assessment on HaCaT and SH-SY5Y cell lines showed the absence of basal toxicity below a 100 µM treatment dose, suggesting these compounds as promising candidates for further exploration in cosmeceutics. The repair capacity of compounds **4b** and **5b** was dose-dependent, and it was similar to that exerted by the corresponding HCAs even at low doses. Considering that a greater cellular uptake was not directly correlated to enhanced wound closure, it could be hypothesized that the mechanism involved the interaction with outer cell membrane constituents. These preliminary but promising results certainly encourage the deepening of the mechanisms of action. In addition, future prospects could involve the development of innovative cosmeceutical formulations to assess their post-topical bioavailability, skin permeation and irritation assessments.

## Data Availability

Data are available within the manuscript and in Supplementary Materials.

## Supplementary Materials

The following supporting information can be downloaded at: https://www.mdpi.com/article/10.3390/antiox13060744/s1, Figure S1: ^1^H-NMR (A) and ^13^C-NMR (B) spectra of compound **3a** in CDCl_3_. Figure S2: ^1^H-NMR (A) and ^13^C-NMR (B) spectra of compound **3b** in CD_3_OD.
