# Supplementary material for: Innovative Cosmeceutical Ingredients: Harnessing Selenosugar-Linked Hydroxycinnamic Acids for Antioxidant and Wound-Healing Properties
Source: Antioxidants (Basel). 2024 Jun 20;13(6):744. doi: 10.3390/antiox13060744 (PMC11200926; doi:10.3390/antiox13060744)
Supplement: Supplementary file 1 [file antioxidants-13-00744-s001.zip › antioxidants-3069419-supplementary.pdf]

Innovative Cosmeceutical Ingredients: Harnessing Selenosugar-Linked Hydroxycinnamic Acids for Antioxidant and Wound-Healing Properties

Giovanna Cimmino [1,2], Mauro De Nisco [3], Simona Piccolella [1,*], Claudia Gravina [1], Silvana Pedatella [2] and Severina Pacifico [1]

[1] Department of Environmental, Biological and Pharmaceutical Sciences and Technologies, University of Campania "Luigi Vanvitelli", Via Vivaldi 43, 81100 Caserta, Italy; giovanna.cimmino@unicampania.it (G.C.); claudia.gravina@unicampania.it (C.G.); severina.pacifico@unicampania.it (S.P.)

[2] Department of Chemical Sciences, University of Napoli Federico II, Via Cinthia 4, 80126 Napoli, Italy;
silvana.pedatella@unina.it

[3] Department of Sciences, University of Basilicata, Via dell'Ateneo Lucano 10, 85100 Potenza, Italy; mauro.denisco@unibas.it

* Correspondence: simona.piccolella@unicampania.it

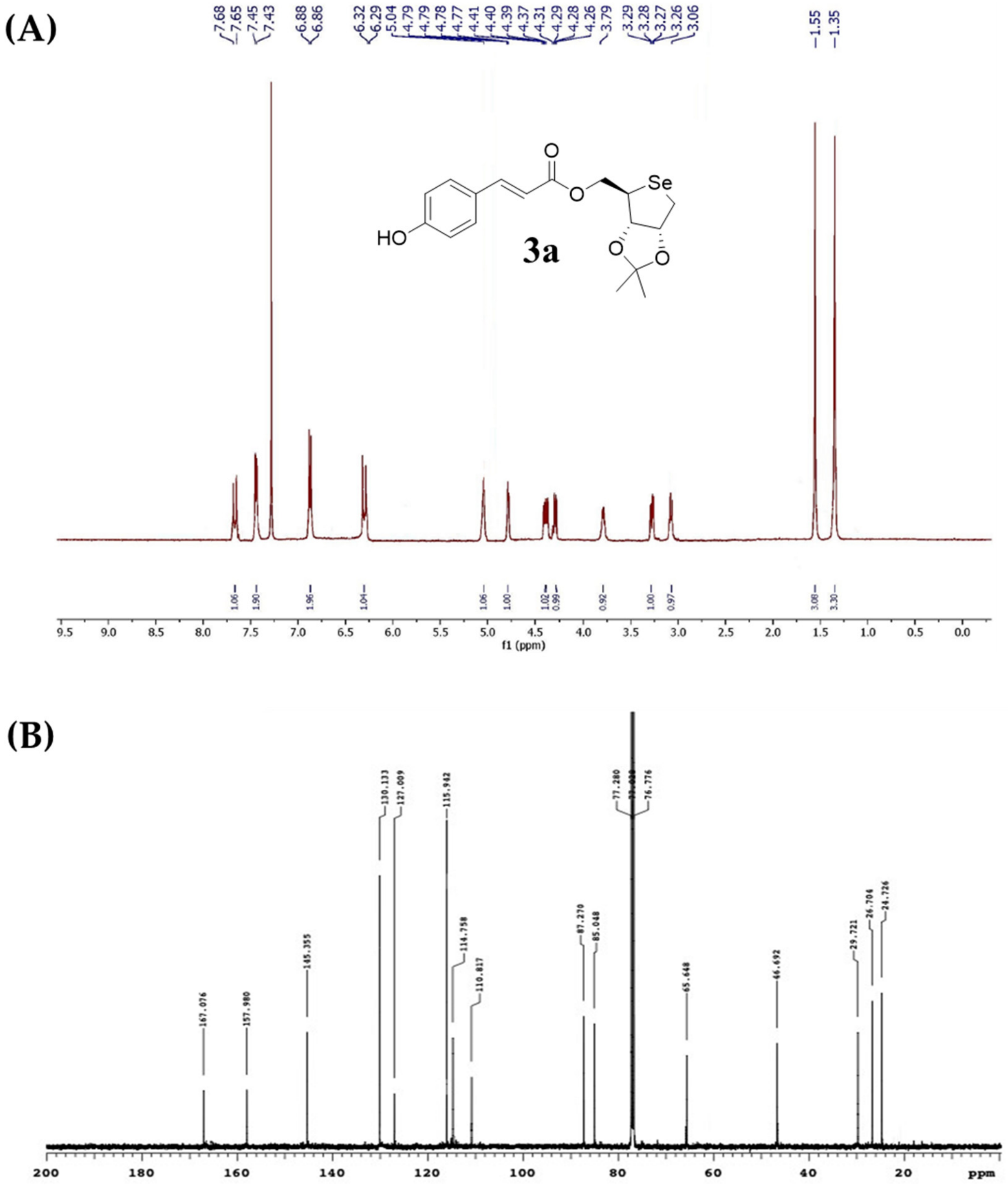

Figure S1. ^{1}H-NMR (A) and ^{13}C-NMR (B) spectra of compound **3a** in $CDCl_3$.

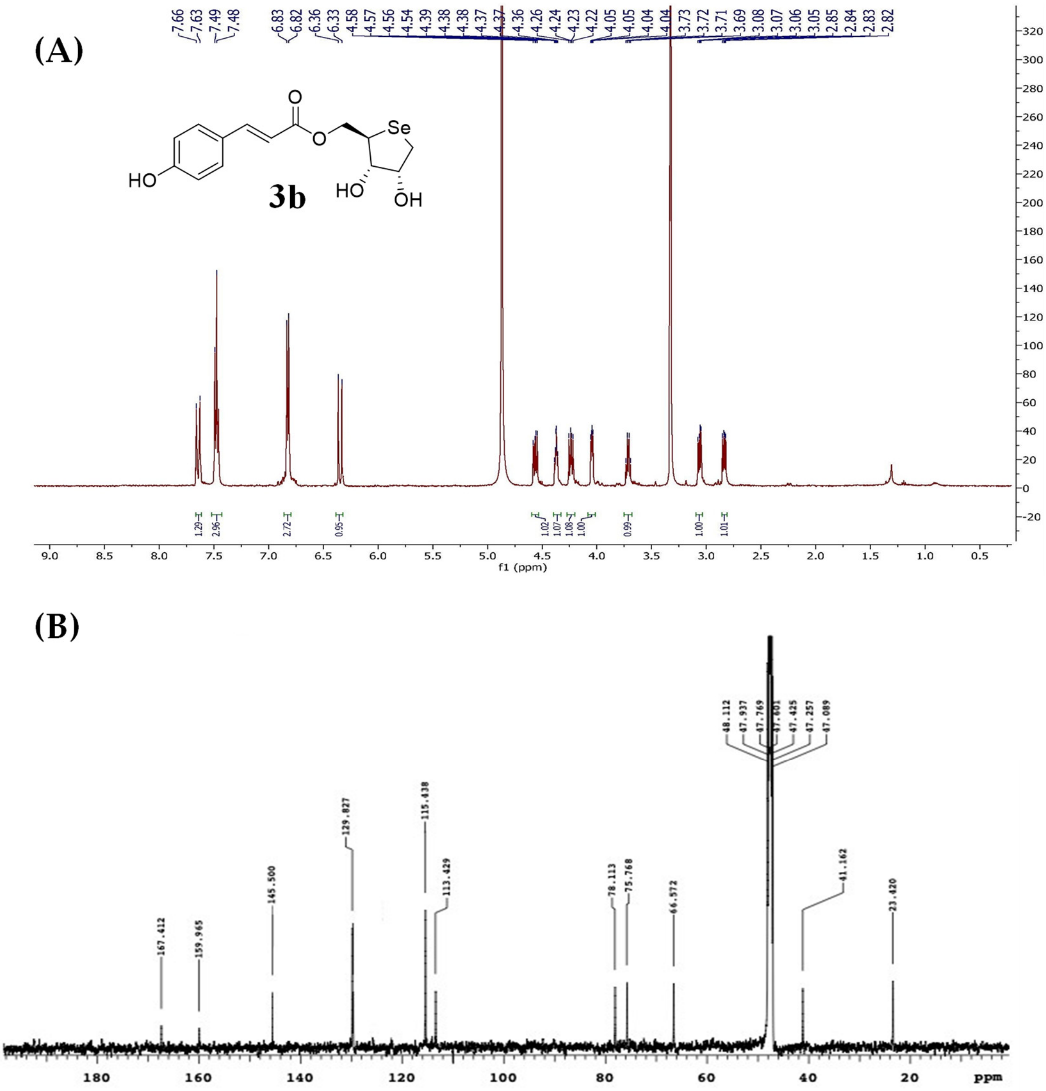

Figure S2. ^{1}H-NMR (A) and ^{13}C-NMR (B) spectra of compound **3b** in CD_3OD.

.
